# Supplementary material for: Finding patterns in lung cancer protein sequences for drug repurposing
Source: PLoS One. 2025 May 7;20(5):e0322546. doi: 10.1371/journal.pone.0322546 (PMC12058034; doi:10.1371/journal.pone.0322546)

**Supporting Information**

**Figure S1.** Histograms show the distribution of the four protein similarity metrics across the different datasets.


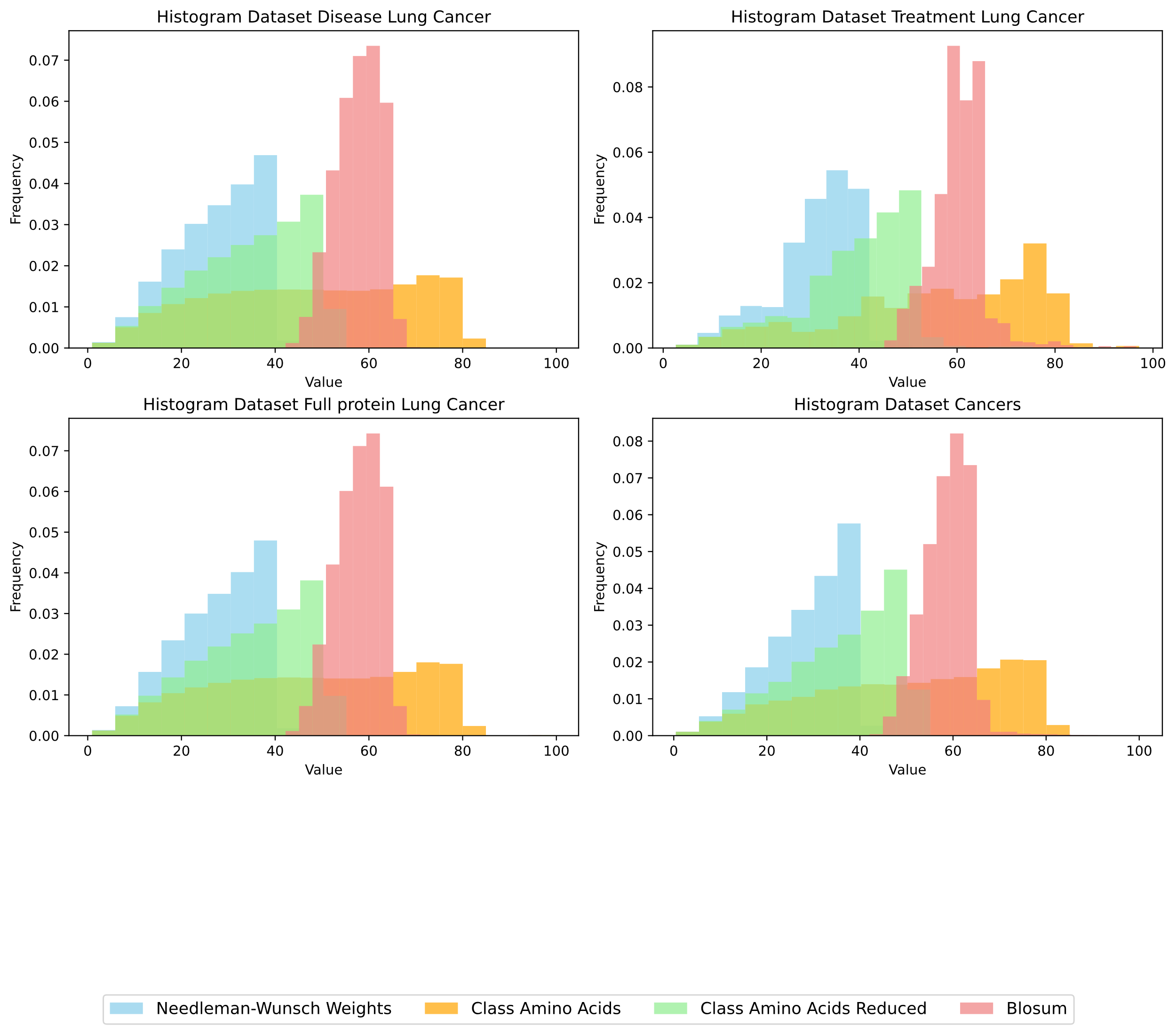

Supplement: S1 Figure — (DOCX) [file pone.0322546.s001.docx]
