# Supplementary material for: Finding patterns in lung cancer protein sequences for drug repurposing
Source: PLoS One. 2025 May 7;20(5):e0322546. doi: 10.1371/journal.pone.0322546 (PMC12058034; doi:10.1371/journal.pone.0322546)
Supplement: S1 File — Likelihood of subsequence occurrence and threshold determination. (DOCX) [file pone.0322546.s002.docx]

**Supporting Information**

**Methods S1: Likelihood of subsequence occurrence and threshold determination**

Proteins sequences are composed of 20 standard amino acids. The number of possible subsequences of length *x* can be determined using the formula:

$${20}^{x}$$

where *x* represents the length of the subsequence. This calculation provides the total number of unique possible combinations for a given subsequence length. For different values of *x*, the number of possible subsequences is as follows:

| **x (subsequence length)** | 1 | 2 | 3 | 4 | 5 | 6 | ... |
| --- | --- | --- | --- | --- | --- | --- | --- |
| **Number of Combinations** | 20 | 400 | 8,000 | 160,000 | 3,200,000 | 64,000,000 | ... |

Assuming that all amino acid combinations are equally probable (i.e., there are no known biochemical restrictions or biases), the likelihood of observing any specific subsequence *s* of length *x* is given by:

$$L(s)=\frac{1}{{20}^{x}}$$

This likelihood represents the chance of encountering a particular subsequence *as purely* by chance. The following table illustrates these likelihoods for different values of *x*:

| **x (subsequence length)** | 1 | 2 | 3 | 4 | 5 | 6 | ... |
| --- | --- | --- | --- | --- | --- | --- | --- |
| **Likelihood of a Specific Subsequence** | 1/20 | 1/400 | 1/8,000 | 1/160,000 | 1/3,200,000 | 1/64,000,000 | ... |

This likelihood calculation provides an expected baseline for how frequently a particular subsequence should appear randomly within a protein sequence. However, as subsequences can be located at any position within a protein, it is essential to consider protein length as well.

To account for protein length, the likelihood of a given subsequence *s* of length *x* appearing in a protein *p* of length *protein_length* is calculated as follows:

$$L(s, p)=1- \left( 1- \frac{1}{{20}^{x}} \right)^{protein\_length - (x-1)}$$

The pattern discovery algorithm was applied to the *lung treatment* dataset, which consists of 52 protein sequences with an average length of 519 amino acids. To evaluate the likelihood of random occurrence for different subsequence lengths, the following table was generated:

| **x (subsequence length)** | **Likelihood of Occurrence in a Protein of Length 519** |
| --- | --- |
| 1 | 0.999999999997255 $\approx$ 100% |
| 2 | 0.7265 $\approx$72.65% |
| 3 | 0.0626 $\approx$6.26% |
| 4 | 0.0032 $\approx$0.32% |
| 5 | 0.0001609 $\approx$ 0.01609% |
| 6 | 0.00000803 $\approx$ 0.000803% |
| ... | ... |

To define significant patterns, we established empirical frequency thresholds based on dataset characteristics. Since our dataset consists of 52 proteins, a 5% threshold corresponds to 3 proteins. This threshold is well above the expected 0.32% likelihood for *x = 4*, ensuring that patterns identified at this level are unlikely to be random.

For *x = 3*, the expected likelihood of random occurrence in a protein of length 519 is 6.26%, which is significantly higher than 5%. This justifies the exclusion of length-3 patterns at the 5% occurrence threshold. Conversely, applying a stricter threshold (e.g., >10%) results in the identification of very few patterns. These thresholds are specific to this dataset and may require adjustment when analyzing different datasets.

The calculations above assume an average protein length of 519 amino acids. However, the *lung treatment* dataset exhibits substantial variability, with a standard deviation of 367 amino acids. The shortest protein in the dataset contains only 66 amino acids, while the longest reaches 2,549 amino acids. This significant variance impacts the likelihood calculations and suggests that length normalization methods may be necessary for future analyses.

This methodology ensures a rigorous statistical approach to identifying significant amino acid patterns while minimizing false discoveries due to random occurrences. By incorporating both combinatorial likelihoods and dataset-specific constraints, we establish a framework that can be adapted for broader applications in protein sequence analysis.
