# Supplementary material for: Finding patterns in lung cancer protein sequences for drug repurposing
Source: PLoS One. 2025 May 7;20(5):e0322546. doi: 10.1371/journal.pone.0322546 (PMC12058034; doi:10.1371/journal.pone.0322546)
Supplement: S1 Table — (DOCX) [file pone.0322546.s003.docx]

**Supporting Information**

**Table S1.** Shows these drugs together with their target protein, the derived gene, and the effect of the drug on the disease.

| **drug_id** | **drug_name** | **action_type** | **protein_id** | **gene_symbol** |
| --- | --- | --- | --- | --- |
| CHEMBL1006 | AMIFOSTINE | INDUCER | P10696 | ALPG |
| CHEMBL1023 | BEXAROTENE | AGONIST | P28702 | RXRB |
| CHEMBL1023 | BEXAROTENE | AGONIST | P48443 | RXRG |
| CHEMBL1023 | BEXAROTENE | AGONIST | P19793 | RXRA |
| CHEMBL109 | VALPROIC ACID | INHIBITOR | P51649 | ALDH5A1 |
| CHEMBL109 | VALPROIC ACID | INHIBITOR | Q92769 | HDAC2 |
| CHEMBL139 | DICLOFENAC | INHIBITOR | P23219 | PTGS1 |
| CHEMBL139 | DICLOFENAC | INHIBITOR | P35354 | PTGS2 |
| CHEMBL159 | VINBLASTINE | ADDUCT | Q71U36 | TUBA1A |
| CHEMBL159 | VINBLASTINE | OTHER | P05412 | JUN |
| CHEMBL159 | VINBLASTINE | UNKNOWN | P05412 | JUN |
| CHEMBL159 | VINBLASTINE | ADDUCT | P07437 | TUBB |
| CHEMBL185 | FLUOROURACIL | INHIBITOR | P04818 | TYMS |
| CHEMBL1908360 | EVEROLIMUS | INHIBITOR | P62942 | FKBP1A |
| CHEMBL1908360 | EVEROLIMUS | INHIBITOR | P42345 | MTOR |
| CHEMBL225072 | PEMETREXED | INHIBITOR | P04818 | TYMS |
| CHEMBL225072 | PEMETREXED | INHIBITOR | P00374 | DHFR |
| CHEMBL225072 | PEMETREXED | INHIBITOR | P31939 | ATIC |
| CHEMBL225072 | PEMETREXED | INHIBITOR | P22102 | GART |
| CHEMBL2362016 | ARSENIC TRIOXIDE | INHIBITOR | Q16881 | TXNRD1 |
| CHEMBL238071 | VINDESINE | INHIBITOR | Q9H4B7 | TUBB1 |
| CHEMBL25 | ASPIRIN | DOWNREGULATOR | P01106 | MYC |
| CHEMBL25 | ASPIRIN | INHIBITOR | P23219 | PTGS1 |
| CHEMBL25 | ASPIRIN | INHIBITOR | P35354 | PTGS2 |
| CHEMBL25 | ASPIRIN | DOWNREGULATOR | P42574 | CASP3 |
| CHEMBL25 | ASPIRIN | INHIBITOR | P42574 | CASP3 |
| CHEMBL25 | ASPIRIN | DOWNREGULATOR | P12004 | PCNA |
| CHEMBL25 | ASPIRIN | ACTIVATOR | Q9UGI9 | PRKAG3 |
| CHEMBL25 | ASPIRIN | DOWNREGULATOR | P24385 | CCND1 |
| CHEMBL25 | ASPIRIN | ACETYLATION | P04637 | TP53 |
| CHEMBL25 | ASPIRIN | INDUCER | P04637 | TP53 |
| CHEMBL25 | ASPIRIN | DOWNREGULATOR | P29466 | CASP1 |
| CHEMBL25 | ASPIRIN | INHIBITOR | P29466 | CASP1 |
| CHEMBL25 | ASPIRIN | INHIBITOR | Q04828 | AKR1C1 |
| CHEMBL254219 | DIGITOXIN | INHIBITOR | P50993 | ATP1A2 |
| CHEMBL254219 | DIGITOXIN | INHIBITOR | P54710 | FXYD2 |
| CHEMBL254219 | DIGITOXIN | INHIBITOR | P05023 | ATP1A1 |
| CHEMBL325041 | BORTEZOMIB | INHIBITOR | Q99460 | PSMD1 |
| CHEMBL325041 | BORTEZOMIB | INHIBITOR | P20618 | PSMB1 |
| CHEMBL325041 | BORTEZOMIB | INHIBITOR | P28074 | PSMB5 |
| CHEMBL34259 | METHOTREXATE | INHIBITOR | P00374 | DHFR |
| CHEMBL34259 | METHOTREXATE | INHIBITOR | P04818 | TYMS |
| CHEMBL34259 | METHOTREXATE | INHIBITOR | P31939 | ATIC |
| CHEMBL408 | TROGLITAZONE | AGONIST | P37231 | PPARG |
| CHEMBL408 | TROGLITAZONE | INHIBITOR | Q99808 | SLC29A1 |
| CHEMBL408 | TROGLITAZONE | INVERSE AGONIST | P11474 | ESRRA |
| CHEMBL408 | TROGLITAZONE | ANTAGONIST | P05121 | SERPINE1 |
| CHEMBL408 | TROGLITAZONE | INVERSE AGONIST | P62508 | ESRRG |
| CHEMBL428647 | PACLITAXEL | INHIBITOR | Q9H4B7 | TUBB1 |
| CHEMBL428647 | PACLITAXEL | INDUCER | O75469 | NR1I2 |
| CHEMBL428647 | PACLITAXEL | INHIBITOR | P10415 | BCL2 |
| CHEMBL468 | THALIDOMIDE | INHIBITOR | P01375 | TNF |
| CHEMBL468 | THALIDOMIDE | ANTAGONIST | P35354 | PTGS2 |
| CHEMBL468 | THALIDOMIDE | INHIBITOR | Q96SW2 | CRBN |
| CHEMBL473417 | VISMODEGIB | INHIBITOR | Q99835 | SMO |
| CHEMBL481 | IRINOTECAN | INHIBITOR | P11387 | TOP1 |
| CHEMBL481 | IRINOTECAN | INHIBITOR | Q969P6 | TOP1MT |
| CHEMBL547 | ISOTRETINOIN | OTHER | P10276 | RARA |
| CHEMBL547 | ISOTRETINOIN | UNKNOWN | P10276 | RARA |
| CHEMBL553 | ERLOTINIB | AGONIST | O75469 | NR1I2 |
| CHEMBL553025 | VINORELBINE | ANTAGONIST | P07437 | TUBB |
| CHEMBL553025 | VINORELBINE | INHIBITOR | P07437 | TUBB |
| CHEMBL56367 | NIMESULIDE | INHIBITOR | P35354 | PTGS2 |
| CHEMBL84 | TOPOTECAN | INHIBITOR | P11387 | TOP1 |
| CHEMBL84 | TOPOTECAN | INHIBITOR | Q969P6 | TOP1MT |
| CHEMBL888 | GEMCITABINE | INHIBITOR | P23921 | RRM1 |
| CHEMBL888 | GEMCITABINE | INHIBITOR | P04818 | TYMS |
| CHEMBL888 | GEMCITABINE | INHIBITOR | P30085 | CMPK1 |
| CHEMBL92 | DOCETAXEL | BINDER | O75469 | NR1I2 |
| CHEMBL924 | ZOLEDRONIC ACID | INHIBITOR | P14324 | FDPS |
| CHEMBL964 | DISULFIRAM | INHIBITOR | P05091 | ALDH2 |
| CHEMBL98 | VORINOSTAT | INHIBITOR | O15379 | HDAC3 |
| CHEMBL98 | VORINOSTAT | INHIBITOR | Q92769 | HDAC2 |
| CHEMBL98 | VORINOSTAT | INHIBITOR | Q13547 | HDAC1 |
