# Supplementary material for: Finding patterns in lung cancer protein sequences for drug repurposing
Source: PLoS One. 2025 May 7;20(5):e0322546. doi: 10.1371/journal.pone.0322546 (PMC12058034; doi:10.1371/journal.pone.0322546)
Supplement: Attachment — Submitted filename: Response to Reviewers.docx [file pone.0322546.s005.docx]

Subject: Response to Reviewers’ Comments on Manuscript [PONE-D-24-57595]

Dear Dr. Khalid Raza,

We appreciate the time and effort that you and the reviewers have taken to evaluate our manuscript titled “Finding patterns in lung cancer protein sequences for drug repurposing”. We are grateful for the constructive feedback which has helped us improve the quality of our work.

We have carefully revised the entire manuscript to address the reviewer’s comments regarding punctuation, spacing, and overall clarity. During this process, we identified some sentences that could be unclear and have improved their readability accordingly. These modifications have been highlighted in the revised manuscript.

Additionally, given that we received questions regarding the selection of the pattern length in the analysis and the occurrence values, we have now included a detailed explanation and calculations in the Supporting Information. We believe this addition will be valuable for readers who may have similar questions. Below, we provide our responses to the editor and reviewer’s comments. All changes in the revised manuscript have been highlighted for easy reference.

Thank you for your comments. We have carefully reviewed the points raised. Please let us know if any additional information or modifications are needed.

***1.*** *Please ensure that your manuscript meets PLOS ONE's style requirements, including those for file naming.*

Regarding point one, we have carefully reviewed the manuscript and have ensured full compliance with all of PLOS ONE's style guidelines, including the correct application of file naming conventions. Additionally, we have taken the necessary steps to remove any zip codes as per the journal's requirements. Furthermore, we have updated the manuscript to include all requested information for the corresponding author, ensuring that every detail aligns with the submission guidelines.

***2.*** *Please note that PLOS ONE has specific guidelines on code sharing for submissions in which author-generated code underpins the findings in the manuscript.*

We would like to confirm that our code and related files have been made publicly available in a repository, ensuring unrestricted access for the scientific community. This ensures full reproducibility and facilitates reuse, fully in accordance with PLOS ONE’s code-sharing guidelines.

***3.*** *We note that the grant information you provided in the ‘Funding Information’ and ‘Financial Disclosure’ sections do not match.*

We have reviewed and corrected the grant information to ensure consistency between the ‘Funding Information’ and ‘Financial Disclosure’ sections. The correct grant number have now been provided.

***4.*** *Thank you for stating the following financial disclosure:*

*[This research was funded by the project “Data-driven drug repositioning applying graph neural networks (3DR-GNN)” (PID2021-122659OB-I00) from the Spanish Ministerio de Ciencia e Innovación, “Drug repurposing hypotheses through a data-driven approach (GRENADA)” (PDC2022-133173-I00) from the Spanish Ministerio de Ciencia e Innovación and MadridDataSpace4Pandemics, funded by Comunidad de Madrid (Consejería de Educación, Universidades, Ciencia y Portavocía) with FEDER funds as part of the response from the European Union to COVID-19 pandemia. Belen Otero Carrasco’s work is supported by “Formación de Personal Investigador” grant (FPI PRE2019-090912) as part of the project “DISNET (Creation and analysis of disease networks for drug repurposing from heterogeneous data sources applied to rare diseases)” (RTI2018-094576-A-I00) from the Spanish Ministerio de Ciencia, Innovación y Universidades.].*

*Please state what role the funders took in the study. If the funders had no role, please state: ""The funders had no role in study design, data collection and analysis, decision to publish, or preparation of the manuscript.""*

*If this statement is not correct you must amend it as needed.*

*Please include this amended Role of Funder statement in your cover letter; we will change the online submission form on your behalf.*

Thank you for your comments. We have amended the financial disclosure statement as requested. We have now added the appropriate statement regarding the role of the funders in the study. The revised statement has been included in the cover letter as per your instructions.

***5.*** *We note that your Data Availability Statement is currently as follows: [All relevant data are within the manuscript and its Supporting Information files.]*

*Please confirm at this time whether or not your submission contains all raw data required to replicate the results of your study. Authors must share the "minimal data set" for their submission. PLOS defines the minimal data set to consist of the data required to replicate all study findings reported in the article, as well as related metadata and methods (*[[*https://journals.plos.org/plosone/s/data-availability#loc-minimal-data-set-definition*](https://journals.plos.org/plosone/s/data-availability#loc-minimal-data-set-definition)](https://journals.plos.org/plosone/s/data-availability#loc-minimal-data-set-definition)*).*

We have reviewed the Data Availability Statement and have updated it accordingly. The relevant data is now available and has been deposited in a public repository, ensuring that the minimal data set required to replicate the study’s findings is accessible.

***6.*** *Please upload a copy of Figure S1 to which you refer in your text on page 34. Please amend the file type to 'Supporting Information'. If the Supplementary file is no longer to be included as part of the submission, please remove all reference to it within the text.*

We have uploaded a copy of Figure S1, as referenced in the text on page 34. Additionally, we have also uploaded the Supporting Information Methods S1 and Table S1. Both files have been correctly marked as “Supporting Information”.

**Reviewers**

**Reviewer #1:** The study is a novel approach of computational methods to identify protein sequence patterns for drug repurposing, specially for NSCLC treatment. The work integrated data from sources, such as UniProt and DISNET, to compile and for analysis and reproducibility. This the study provides new idea for drug repurposing and to understand protein functionality. Further, validation through 3D structural analysis using AlphaFold and ChimeraX adds robustness to the findings, to bridge the computational and structural biology. The step-by-step description of pattern discovery, statistical analysis, and similarity metrics shows the clarity. However the limitations include:

**R1 – C1:** *No laboratory experiments to validate.*

Thank you for this important point. This study is based on a fully computational approach, which serves as a crucial preliminary step before experimental validation. By refining our methodology in this phase, we aim to generate valuable insights that can guide future laboratory experiments. While direct experimental validation is not included in the present work, we recognize its importance and are actively initiating collaborations with researchers who have conducted relevant laboratory experiments in this area. These collaborations will enable us to explore whether our hypotheses align with experimental findings, thereby enhancing the robustness and applicability of our results. Additionally, the data and results presented in this work can serve as a valuable resource for other researchers conducting similar analyses, facilitating knowledge exchange and future experimental studies.

**R1 – C2:** *Sensitivity analysis for varying thresholds is not discussed*

Thank you for your valuable comment. We understand sensitivity analysis as the process of testing how changes in key parameter (in this case, thresholds), impact the outcome. We address this explanation of the thresholds used, 5% and 10% in comment R1-C3. If the comment refers to the similarity calculations, we would like to clarify that the four metrics were computed, and the statistical analysis was conducted following the establish methodology in this field.

**R1 – C3:** *Missing statistical justification for pattern selection, especially the minimum length of four amino acids and occurrence rates.*

In the context of our research, a pattern is a common subsequence that appears in a significant number of sequences within a group. In this study, we propose identifying patterns within a group of NSCLC treatment protein sequences and subsequently checking whether any of these patterns appear in protein sequences associated with other diseases. Due to natural variations in proteins, patterns can arise by chance. However, this randomness diminishes when considering related proteins that share common structural and functional traits [Ref 1].

The total number of possible subsequences is proportional to the subsequence size (length) and the number of available elements for combination. In protein sequences, these elements correspond to the 20 basic amino acids, where each amino acid can occupy any position within the sequence. The number of possible distinct subsequences of these elements is computed as a variation with repetition and is given by Equation 1, where x denotes the length of the subsequence:

$$\mathrm{VR}_{20,x}={20}^{x} (1)$$

As x increases, the likelihood of identifying a recurring pattern decreases, meaning that longer subsequences are less likely to appear in a sufficiently high number of proteins. Given that amino acid combinations are assumed to be equally likely, the likelihood of a given subsequence is $\frac{1}{{20}^{x}}$ . Additionally, these subsequences can emerge at any position within a protein sequence. Accordingly, to test whether a given subsequence of length x appears in a protein of length protein_length, considering that any position up to the last (x-1) is a potential place for it to be located, the number of chances for that subsequence to occur is therefore protein_length - (x - 1).

The choice of a minimum pattern length of four amino acids is based on both probabilistic and biological reasoning. Since protein sequences are composed of 20 amino acids, the number of possible distinct subsequences increases exponentially with length. For example, there are 160,000 possible combinations for sequences of length 4, while for sequences of length 6, this number grows to 6,400,000. Given that amino acid combinations are assumed to be equally likely, shorter patterns have a much higher likelihood of appearing randomly within a dataset.

This effect can be quantified using probability calculations. When analyzing protein sequences, a pattern of length 4 has a significantly higher chance of emerging by chance than longer patterns. In contrast, as the length of the pattern increases, the probability of random occurrence decreases exponentially, making these longer patterns much less likely to appear unless they have functional or structural significance. This is particularly relevant in our dataset, where the treatment set consists of only 52 proteins. In such a small dataset, even detecting patterns of length 4 is already challenging unless these patterns are genuinely shared among proteins rather than appearing due to random chance.

From a biological perspective, shorter patterns tend to carry less specific information about structural or functional properties of proteins. Since amino acids interact in groups to define motifs, domains, and binding sites, patterns of only two or three amino acids may not be sufficient to capture relevant biological features, as they can be part of many unrelated sequences. As pattern length increases, there is a greater likelihood that these sequences correspond to conserved or functionally relevant regions, making them more informative for understanding protein interactions and mechanisms.

For this reason, setting a minimum threshold of four amino acids ensures that the identified patterns are less likely to be artifacts of random sequence variability and increases the probability of capturing biologically relevant motifs. Additionally, previous studies have established that biologically relevant sequence similarities are typically observed for subsequences of at least four amino acids [Ref 2]. This threshold is thus a compromise between statistical robustness and biological interpretability, allowing us to identify meaningful patterns while minimizing the likelihood of coincidental similarities.

The occurrence rates represent the number of proteins that must share a pattern for it to be considered relevant. In this study, we employed 5% and 10% occurrence thresholds, which act as hyperparameters that regulate the balance between specificity and generality in pattern detection. A higher threshold (10%) is more restrictive, leading to the identification of shorter, more conserved patterns, as it requires them to be present in a greater number of proteins. Conversely, a lower threshold (5%) is more permissive, allowing for the detection of longer patterns that appear in fewer proteins, which suggests they may be more selective or associated with specific subgroups within the dataset.

The selection of these thresholds was driven by empirical evaluation, ensuring that the identified patterns were sufficiently long (at least four amino acids) while still being general enough to capture relevant sequence information. Through iterative testing, we observed that 5% and 10% provided an optimal balance between detecting meaningful patterns and avoiding excessive variability. Values above 10% significantly reduced the number of detected patterns, potentially discarding relevant information, while values below 5% introduced too much variability, leading to patterns that were less reproducible or less biologically meaningful. Additionally, three-dimensional structural analysis supported the biological relevance of the identified patterns, particularly for proteins within the same family or functionally related groups.

It is important to emphasize that these thresholds are dataset-dependent and should not be considered fixed for all studies. The optimal occurrence rate must be adjusted based on the nature and size of the dataset to ensure the best balance between specificity and sensitivity. Future applications of this methodology should consider tuning this hyperparameter according to the characteristics of the protein sequences under investigation, as different datasets may require different thresholds to maximize biological interpretability.

To address these concerns, an annex has been created and will be included in the supporting information (Methods S1). The formulas applied to our dataset yielded the results that are provided in the supporting information containing the calculations and tables. This has been provided to the reviewer.

[Ref 1] - Z. Zou & J. Zhang. 2019 Amino acid exchangeabilities vary across the three of life, Sci. Adv., vol. 5, no. 12, p. eaax3124.

[Ref 2] - M. A. Roytber. 1992. A search for common patterns in many sequences. *CABIOS*, vol. 8, no. 1, pages: 57-64.

**R1 – C4:** *The RMSD there is no detailed explanation of how deviations impact biological functionality.*

Thank you for highlighting this point. Various methods are regularly used to compare computational models with experimental data in modeling assessments. Among these, RMSD is the most applied quantitative metric for evaluating the similarity between two superimposed atomic coordinate sets, typically expressed in angstroms. Global RMSD, which is based on positional distance, is often used to assess overall protein structure similarity. The RMSD value represents the average deviation between corresponding atoms of two proteins, with lower RMSD indicating greater structural similarity. The primary drawback of RMSD is that it is heavily influenced by the size of errors. Two structures that are otherwise identical, except for the position of a single loop or a flexible terminus, often show a large global backbone RMSD [Ref 1]. It has been shown through analyses evaluating protein models against native structures that RMSD exhibits the greatest variation. RMSD values are significantly influenced by large errors, while other scores tend to focus on more accurate regions or are inherently local. As a result, RMSD is the least reliable in this context, as incomplete models are more likely to receive better scores [Ref 2]. In the present study, RMSD was used as a metric to evaluate the differences between the protein linked to a drug (treatment) and the candidate. Since the goal was to quantify structural differences, RMSD was chosen to determine how similar or diverse the evaluated proteins were.

[Ref 1] - Kufareva, I. & R. Abagyan R. 2012. Methods of protein structure comparison. Methods *Mol Biol* 857: 231−257

[Ref 2] - Olechnovic, K., Monastyrskyy, B., Kryshtafovych, A., and C. Venclovas. 2019. Comparative analysis of methods for evaluation of protein models against native structures. *Bioinformatics*, 35(6), 937–944.

**Reviewer #2:**

**R2 – C1:** *How did the amino acid sequences for each lung cancer drug target protein get gathered in order to find important trends in the protein sequences of treatments?*

The amino acid sequences for each lung cancer drug target protein were obtained from UniProt, a comprehensive protein sequence database. These sequences were then integrated into DISNET, a platform that aggregates biomedical data, enabling a systematic analysis of disease-related proteins and their interactions.

Within DISNET, the dataset was filtered to include only therapeutic drugs targeting lung cancer proteins, following the classification criteria of the Clinical Drug Target (CDT) framework. This approach ensured that only relevant drug-target interactions were considered, allowing researchers to identify key trends in protein sequences associated with effective lung cancer treatments. By leveraging these structured datasets, patterns in sequence conservation, functional domains, and pharmacological relevance were systematically analyzed, contributing to a deeper understanding of targeted therapies.

**R2 – C2:** *Why was the DISNET database picked to store the amino acid sequences of proteins*

*that are used to treat lung cancer? What does DISNET do better than other databases?*

DISNET was selected as the database for storing amino acid sequences of protein used in lung cancer treatment due to its ability to systematically extract, integrate, and structure disease-related knowledge from multiple public sources [Ref 1]. Unlike traditional protein or disease databases, DISNET provides a comprehensive, multi-layered framework that organizes information into three distinct layers:

- Biological layer: Contains information about diseases and their relationships with various biological entities, such as genes and proteins.
- Phenotypical layer: Maps diseases to their associated symptoms.
- Pharmacological layer: Stores data on drugs and their interactions with diseases.

By integrating these layers within a single, structured repository, DISNET facilitates the development of computational experiments with a more holistic perspective.

What sets DISNET apart is its unique ability to interconnect diseases based on shared symptoms and diagnostic terms, enabling a broader and more insightful analysis of disease relationships. Additionally, its freely accessible API allows for customizable data extraction and seamless integration into diverse research workflows. Another key advantage is its capability to track changes in medical knowledge over time, which is particularly valuable in fields like cancer research, where biomarkers and treatment strategies continuously evolve.

These features make DISNET a powerful and dynamic tool for storing and analyzing protein sequences relevant to lung cancer therapy. Moreover, DISNET has been the primary database developed and maintained by the authors in recent years, making it a natural and well-established reference for this study, further reinforcing its relevance as a primary data source.

[Ref 1] - Lagunes-García, G., Rodríguez-González, A., Prieto-Santamaría, L., García Del Valle, E. P., Zanin, M., & E. Menasalvas-Ruiz. 2020. DISNET: a framework for extracting phenotypic disease information from public sources. *PeerJ*, *8*, e8580.

**R2 – C3:** *Refine the loops of the proteins shown in Figures 10 and 11.*

Thank you for your suggestion. We have updated the figures with a white background and enhanced the shape definition, improving the overall clarity. These changes provide a clearer overview of the loops in Figures 10 and 11.

**R2 – C4:** *The RMSD values are usually between 0–2Å, but Table 3 shows unusually high RMSD values for the target structures compared to the reference.*

We thank the reviewer for the valuable suggestion. A common method for comparing predicted and experimentally determined structures (from X-ray, NMR, or EM techniques) is to compute the RMSD between them. This value represents the average deviation between corresponding atoms of the two proteins, with a lower RMSD indicating greater structural similarity. The variability in RMSD values, which is influenced by the selection of proteins sharing the detected patterns, was analyzed by comparing the predicted protein structure generated by AlphaFold. This emphasizes the unique characteristics of the proteins being studied. In ChimeraX, the RMSD command calculates the root-mean-square deviation (RMSD) between two sets of atoms in their current positions, without applying any fitting. RMSD values are high because the structures of different proteins are being compared, which only share similar patterns. These calculations help us contextualize the results observed through 3D visualization. Previous studies have suggested that calculating the RMSD between a protein and its orthologs (using one as a reference structure against the matching structure) could provide more insights into their overall variations [Ref 1]. The same method is applied here between the target protein and the one from the treatment dataset.

[Ref 1] - Tejera-Nevado, P., E. Serrano, A. González-Herrero, R. Bermejo and A. Rodríguez-González. 2024. Unlocking the power of AI models: exploring protein folding prediction through comparative analysis. *Journal of Integrative Bioinformatics*; 21(2): 20230041.

**R2 – C5:** *Only one amino acid pattern, AALET, has an RMSD score of 4.287.*

Thank you for your insight comment. This response serves as a continuation of our previous discussion, with an extension addressing an important aspect of the RMSD calculations. Specifically, we would like to emphasize that in our study, the proteins used for RMSD calculation were selected from different sequences, derived from predictions. RMSD serves primarily as a tool to compare the structural similarity between distributions of predicted protein structures, rather than as an absolute measure of similarity between identical or closely homologous proteins. This distinction is crucial, as it highlights the role of RMSD in assessing the accuracy of structural predictions relative to expected variations in predicted models, even when the sequences themselves may not be identical. The root mean square deviation (RMSD) is a key measure of protein structure similarity, and predicting protein structures with a low RMSD is highly challenging. A previous study analyzed RMSD distributions and estimated the number of randomly generated protein-like structures required to achieve a specific RMSD relative to the native structure. The findings indicate that obtaining a structure within 6 Å RMSD by chance is highly improbable. Consequently, when a prediction reaches this level of accuracy, it should be regarded as a significant achievement in protein structure prediction [Ref 1].

[Ref 1] - Reva, B. A., A. V. Finkelstein and J. Skolnick. 1997. What is the probability of a chance prediction of a protein structure with an rmsd of 6 Å. *Fold Des*; 3(2): 141-147.

**R2 – C6:** *Increase the resolution of Figure 12 to 300 dpi.*

We appreciate the reviewer’s attention to detail and the suggestion to enhance the quality of Figure 12. In response, we have updated the figure to a resolution of 300 dpi to improve clarity and ensure better visualization.

**R2 – C7:** *Update the graphical abstract to better reflect the key points of the manuscript.*

Thank you again for your insightful suggestion. We understand the importance of ensuring that the graphical abstract effectively conveys the key points of the manuscript. Accordingly, we have updated the graphical abstract to better reflect the main contributions and findings of our study. The revised version is now included in the manuscript.

**R2 – C8:**  *Add a clear and concise takeaway message in the conclusion section.*

Thank you for the suggestion, we acknowledge this point and have made the necessary changes. The conclusion section has been rewritten to make it clearer and more concise. The new version is more structured and includes the following information: (1) summary, (2) relevance of the study, (3) future work, and (4) limitations.

“In summary, our research presents a robust computational methodology for identifying significant protein patterns in disease-related sequences, revealing potential therapeutic targets for conditions such as cancer and autoimmune disorders. This approach also enables the exploration of complex protein relationships across diseases and introduces an innovative strategy for drug repurposing, accelerating treatment discovery.

The detection of patterns in amino acid sequences to identify similar proteins and predict their 3D structures has applications in various fields, including target identification, antibody design, enzyme development, and protein engineering. It plays a crucial role in drug discovery, enabling drug repurposing and therapeutic design, as well as in biotechnology and synthetic biology, where protein functionality can be modified or enhanced.

Future work will focus on expanding the methodology to additional diseases, assessing the biological relevance of identified patterns, and visualizing 3D structures to better understand their role in drug interactions. These findings may also inform new clinical trials, further advancing therapeutic development.

While there are inherent limitations in any in silico approach, such as the absence of drug interaction data, pathway alterations, and expression information, this study establishes a solid foundation for understanding protein relationships and guiding further in this field.”

**R2 – C9:** *Use a white background for the visuals.*

We appreciate the reviewer's suggestion on the background colour of the images. Following this recommendation, we have carefully revised and enhanced all figures to improve their clarity and overall presentation. Figures 10 and 11 have been replaced with updated versions that features a white background, enhancing their clarity.

**R2 – C10:** *After applying the 10% occurrence level, there are only 2,034 unique patterns, which is a lot less than the previous case, even though there are 14,330 pattern-protein combinations.*

We sincerely appreciate the reviewer’s insightful comment on this aspect, as it highlights an important point regarding the relationship between the occurrence threshold and the number of identified patterns. When the 10% occurrence level is applied, it is specifically for identifying patterns in NSCLC drug target proteins. A higher occurrence threshold naturally results in fewer specific patterns, as many lower-frequency patterns are filtered out. The number of patterns obtained at the 10% level is lower than at the 5% level, and a significant portion is discarded because they do not meet the occurrence threshold. Since the 5% threshold is less restrictive, the patterns identified at the 10% level tend to be shorter and may capture less comprehensive sequence information for further analysis. It is also important to note that some highly specific patterns may not reach the 10% threshold simply because they appear in a limited subset of proteins, despite their potential biological relevance.

**R2 – C11:**  *How were the patterns that showed up in proteins linked to NSCLC that were 5% and 10% of the time in lung cancer medicines looked at to find out where they were found? How was this analysis done? What factors or methods were used?*

Thank you for your insightful comment. The analysis of patterns linked to NSCLC in lung cancer medicines was conducted using occurrence thresholds of 5% and 10%. These thresholds represent the minimum proportion of proteins that must share a sequence regularity for it to be considered a relevant pattern.

The process began with an exploratory phase in which initial thresholds of 20% and 15% were tested. However, the patterns identified at these levels were not considered meaningful for further analysis, as they were too restrictive and did not capture relevant sequence information. Based on these observations, the occurrence rate was treated as a hyperparameter, allowing us to optimize pattern detection by balancing specificity and generalizability.

At a 10% threshold, the identified patterns were generally shorter and more specific, appearing in a limited number of proteins. In contrast, the 5% threshold—being less restrictive—produced longer patterns, as it allowed for the identification of more widespread sequence regularities. However, while the 5% threshold captured additional information, these patterns were not necessarily specific to a single protein.

The final selection of 5% and 10% as the optimal occurrence thresholds was based on the need to:

1. Identify patterns that were at least four amino acids long to ensure biological relevance.
2. Capture meaningful sequence information without being overly generic.
3. Ensure that the detected patterns corresponded to proteins belonging to the same family or functionally related groups, an observation that was later validated through three-dimensional structural analysis.

By fine-tuning the occurrence threshold, we were able to extract biologically significant sequence patterns relevant to NSCLC-related proteins. While 5% and 10% proved to be the optimal thresholds for this study, future research may require adjusting this hyperparameter based on the specific nature of the dataset under consideration.

**R2 – C12:** *How were the 2,200 patterns found in proteins of different types of cancer, out of the 2,368 patterns found with a 5% chance of occurring in lung cancer treatments? What method or approach was used to find out if they were there?*

Thank you for your thoughtful question. We appreciate the opportunity to clarify the methodology used in this analysis. The identification of the 2,200 patterns in proteins associated with different types of cancer was conducted using a systematic regular expression-based search. Initially, the analysis focused on detecting patterns in NSCLC drug target proteins, identifying recurring motifs that tend to be associated with treated proteins. This approach is based on the premise that similar sequence patterns may serve as potential targets for therapeutic interventions or may be indicative of proteins more likely to be targeted by treatments. To extend the analysis beyond NSCLC, the identified patterns were then searched across datasets of proteins associated with other cancer types. To ensure meaningful results, these disease-related datasets were carefully filtered to exclude any proteins that were already present in the NSCLC treatment dataset used for pattern discovery. This filtering step was crucial, as including such proteins would artificially reinforce the presence of patterns already known to exist in the treatment set, thus diminishing the biological significance of the findings. By applying this methodology, we ensured that the identified patterns were genuinely present in proteins from different types of cancer and not merely a reflection of prior knowledge from NSCLC treatment datasets.

**Reviewer #3:**

The authors propose a computational analysis for identifying potentially drugs candidate for non-small cells lung cancer based on the identification of characteristic patterns that link the drug’s target proteins to the proteins associated to lung cancer. These distinctive patterns of lung cancer drug target proteins were then searched for in other diseases-associated proteins. The manuscript is well-written, scientifically sound, and addresses a relevant topic to the scientific community, but some improvements and clarifications need to be performed to the presentation of the study, especially advances with respect to other DR approaches.

**R3 – C1:** *The study described in the manuscript is really interesting, but the novelty of the method should be better highlighted. The authors should better emphasize the significant advance of this study within the field of network-based strategies for drug repurposing opportunities with respect to other previous published ones about the same topic. In particular, the authors should deeply discuss the:(1) the rationale behind the idea of using disease-related protein sequences and of this approach.(2) the advances of their approach respect to the framework of well-established Network Medicine to find repurposable drugs applied for study for example COVID-19 here https://www.pnas.org/doi/10.1073/pnas.2025581118 and here https://doi.org/10.1371/journal.pcbi.1008686, and in human cancer here:* [*https://www.sciencedirect.com/science/article/pii/S2001037022005311*](https://www.sciencedirect.com/science/article/pii/S2001037022005311)

We sincerely appreciate the reviewer’s insightful comments and their recognition of the relevance of our study. We understand the importance of clearly highlighting the novelty of our approach and its contributions to the field of drug repurposing.

Regarding the rationale behind the use of disease-related protein sequences, our methodology is based on the premise that proteins play essential roles in biological functions and regulation, and alterations in their sequences are often associated with disease mechanisms. By identifying specific sequence patterns within drug target proteins for NSCLC, we aim to uncover biologically relevant motifs that may be shared with proteins linked to other diseases. These patterns, which consist of short amino acid sequences, can be crucial for protein function and interaction, providing a novel perspective for establishing potential therapeutic connections. Unlike traditional network-based drug repurposing approaches, which often rely on protein-protein interaction networks or pathway analyses, our method directly examines sequence-level characteristics. This allows us to identify conserved motifs that may be critical for drug binding or disease association, offering a complementary and sequence-driven strategy for drug repurposing. By applying this methodology, we have successfully identified relationships between NSCLC drug target proteins and proteins from other cancer types, which were further validated through literature-based evidence.

Furthermore, our study differs from these approaches by focusing on the intrinsic sequence characteristics of drug target proteins rather than their network connectivity. Instead of relying on known interactions or associations, we identify conserved sequence motifs within NSCLC drug target proteins and investigate their presence in proteins associated with other diseases. This sequence-driven methodology provides a complementary strategy to traditional network-based approaches, as it highlights potential functional similarities that may not be directly observable through interaction-based analyses.

Additionally, while network-based approaches often require extensive curated databases and known interactions, our methodology can operate independently of such constraints, making it particularly useful for uncovering previously uncharacterized relationships. By integrating this approach with existing network-based frameworks, future studies could potentially enhance drug repurposing strategies by combining interaction-based and sequence-based insights.

**R3 – C2:** *Moreover, these recent studies about successful network-based approaches for drug repurposing applied to study complex diseases, should be cited (for example at line 77, when talking about computational approaches for DR):*

*o Network medicine framework for identifying drug-repurposing opportunities for COVID-19 PNAS May 11, 2021 118 (19) e2025581118; https://doi.org/10.1073/pnas.2025581118*

*o Comprehensive network medicine-based drug repositioning via integration of therapeutic efficacy and side effects. npj Syst Biol Appl 8, 12 (2022). https://doi.org/10.1038/s41540-022-00221-0*

*o SAveRUNNER: an R-based tool for drug repurposing. BMC Bioinformatics 22, 150 (2021). https://doi.org/10.1186/s12859-021-04076-w*

*o Drug Repurposing: A Network-based Approach to Amyotrophic Lateral Sclerosis. Neurotherapeutics. 2021 Jul;18(3):1678-1691. doi: 10.1007/s13311-021-01064-z. Epub 2021 May 13. PMID: 33987813; PMCID: PMC8609089.*

Thank you very much for your valuable comments and suggestions. We have carefully considered the recommendations you provided, and they have been incorporated into the text accordingly. The following sentence has been added, which intrudes this methodology “Among the most promising computational strategies for drug repurposing, network-based approaches have gained significant attention due to their ability to model complex interactions between drugs, targets, and disease pathways. Recent studies have demonstrated the effectiveness of these methods in identifying potential therapeutic agents by leveraging network topology and multi-omics data integration [8–11]. Their application has provided relevant insights into disease mechanisms and drug action, complementing other computational methodologies.”

**R3 – C3:** *The authors could exploit the Connectivity Map (CMAP) database (https://clue.io/) to query for a core set of 9 different cell lines (at https://clue.io/query) in which the compounds and genetic perturbagens have been profiled and to see if the identified drug could conteract the action of the disease signature.*

Thank you for your thoughtful comment. We appreciate your suggestion and would like to clarify that we have been exploring the CLUE platform to see if we could retrieve information through queries based on both drugs and gene sets. This is an avenue we plan to consider in the future, especially as we gain more knowledge and can better assess potential effects on gene expression.

However, for the scope of this study, our primary focus was to determine whether we could identify closely related proteins by recognizing the patterns they share. Our methodology begins with the linear amino acid sequence and extends through to the 3D structure. While further analyses could be conducted in the future to explore potential drug targets and other effects, our current work is concentrated on protein similarity based on these patterns.

We hope this clarification helps, and we look forward to exploring these additional aspects as our research progresses.

**R3 – C4:** *All figures have to be improved. Just few examples:*

*- Fonts are too small and barely readable (e.g. Figure 6, Figure 9) or label appear stretched (Figure 13)*

*- The number of Figure is too high, I suggest to move some figure as supplementary figures.*

Thank you for your valuable feedback. We have improved all the figures as recommended by increasing the font size, enhancing readability, and ensuring better visual clarity. These adjustments make the figures more accessible and easier to interpret.

Regarding the suggestion to remove some figures, we have carefully considered this point but have decided to keep them, as we believe they are important for a clear understanding of the text and contribute to the overall purpose of the article.

**R3 – C5:** *The authors need to include a final conclusion at the end of the manuscript and also discuss about the limitations of their approach.*

We appreciate the reviewer’s valuable feedback. Following the suggestion, we have revised the conclusion section and incorporated the relevance of the study and the limitations of our approach. The updated section is now more structured and includes the following key elements: (1) summary, (2) relevance of the study, (3) future work, and (4) limitations.

“In summary, our research presents a robust computational methodology for identifying significant protein patterns in disease-related sequences, revealing potential therapeutic targets for conditions such as cancer and autoimmune disorders. This approach also enables the exploration of complex protein relationships across diseases and introduces an innovative strategy for drug repurposing, accelerating treatment discovery.

The detection of patterns in amino acid sequences to identify similar proteins and predict their 3D structures has applications in various fields, including target identification, antibody design, enzyme development, and protein engineering. It plays a crucial role in drug discovery, enabling drug repurposing and therapeutic design, as well as in biotechnology and synthetic biology, where protein functionality can be modified or enhanced.

Future work will focus on expanding the methodology to additional diseases, assessing the biological relevance of identified patterns, and visualizing 3D structures to better understand their role in drug interactions. These findings may also inform new clinical trials, further advancing therapeutic development.

While there are inherent limitations in any in silico approach, such as the absence of drug interaction data, pathway alterations, and expression information, this study establishes a solid foundation for understanding protein relationships and guiding further in this field.”

**R3 – C6:** *Minor comments: 1. In general, the authors should check the form of the entire paper avoiding careless errors, missing commas, missing space between words.*

Thank you for your insightful feedback. We have meticulously reviewed the entire manuscript to correct any typographical inconsistencies, including punctuation and spacing issues.

**R3 – C7:** *Minor comments: 2. Abstract is too technical, please avoid acronyms and details that are not useful for fluent and clear reading. Moreover, first sentence is too long, please break it into two sentences or re-formulate it.*

Thank you for your valuable feedback. Taking your suggestions into account, the abstract has been revised to improve clarity and readability.

“Proteins are fundamental biomolecules composed of one or more chains of amino acids. They are essential for all living organisms, contributing to various biological functions and regulatory processes. Alterations in protein structures and functions are closely linked to diseases, emphasizing the need for in-depth study. A thorough understanding of these associations is crucial for developing targeted and more effective therapeutic strategies.

Computational analyses of biomedical data facilitate the identification of specific patterns in proteins associated with diseases, providing novel insights into their biological roles. This study introduces a computational approach designed to detect relevant sequence patterns within proteins. These patterns, characterized by specific amino acid arrangements, can be critical for protein functionality. The proposed methodology was applied to proteins targeted by drugs used in lung cancer treatment, a disease that remains the leading cause of cancer-related mortality worldwide. Given that non-small cell lung cancer represents 85–90% of all lung cancer cases, it was selected as the primary focus of this study.

Significant sequence patterns were identified, establishing connections between drug-target proteins and proteins associated with lung cancer. Based on these findings, a novel computational framework was developed to extend this pattern-based analysis to proteins linked to other diseases. By employing this approach, relationships between lung cancer drug-target proteins and proteins associated with four additional cancer types were uncovered. These associations, characterized by shared amino acid sequence features, suggest potential opportunities for drug repurposing. Furthermore, validation through an extensive literature review confirmed biological links between lung cancer drug-target proteins and proteins related to other malignancies, reinforcing the potential of this methodology for identifying new therapeutic applications.”

**R3 – C8:** *Minor comments: 3. Check all the commas. Add a comma before “and” whenever more than two elements have been listed.*

Thank you for your suggestion. We have carefully reviewed the manuscript and added commas before "and" in lists with more than two elements, as recommended.

**R3 – C9:** *Minor comments: 4. Figure 3’s legend is too long*

Thank you for your comment. We have shortened the legend for Figure 3 as suggested. The revised version is now included in the manuscript, and the updated legend is presented below: “The algorithm is designed to identify patterns in the treatment dataset. This process involves two key functions. First, from lines 1 to 8, the algorithm identifies amino acids that meet the occurrence threshold, starting with patterns of length 1. It then iteratively extends these potential patterns by adding amino acids to previously identified patterns of maximum length. From lines 9 to 21, the algorithm uses the previously found maximum-length patterns to identify those that continue to meet the occurrence criteria and eliminates those that are subpatterns of newly extended patterns (lines 19-21).”

We believe that the revisions have significantly improved our manuscript, and we appreciate the reviewers’ and the editor’s effort in enhancing the quality of our work. Please let us know if any further modifications are required.

Thank you for your consideration.

Sincerely,

Belén Otero Carrasco
